# Research progress on multispecies animal models of chronic rhinosinusitis: modeling strategies and research applications

**DOI:** 10.3389/falgy.2026.1832889

**Published:** 2026-06-03

**Authors:** Minghao Li, Dongdong Zhu, Cuida Meng

**Affiliations:** 1 Department of Otolaryngology Head and Neck Surgery, China-Japan Union Hospital of Jilin University, Changchun, China; 2 Jilin Provincial Key Laboratory of Precise Diagnosis and Treatment of Upper Airway Allergic Diseases, Changchun, China; 3 Otolaryngology Head and Neck Surgery Research Center, Changchun, China

**Keywords:** animal models, chronic rhinosinusitis, immune mechanisms, modeling methods, research applications

## Abstract

Chronic rhinosinusitis (CRS) is a systemic, complicated, inflammatory disease which results in a significant burden on the patient's quality of life. Although the prevalence has been increasing worldwide, our therapeutic advances have been limited by the complexity of immune imbalance in addition to epithelial breakdown. Although murine models have always been the default of choice of mechanistic studies because of their relative affordability and genetic tractability, they are often insufficient to reflect the anatomical and physiological reality of the human nasal sinus. As an example, the sheep model provides a much more convincing explanation of the process of human sinus drainage, and porcine models offer a specialized perspective on the recalcitrant epithelial remodeling that characterizes severe CRS. In this review we go further than the listing of the species. Rather we focus on evaluating the efficiency of these models in representing specific human phenotypes, such as from eosinophilic overproliferation in type 2 inflammation to phenotypes in non-type 2 diseases. Through a new strategic decision framework, we would guide researchers out of paradigms of models of convenience, to models of clinical relevance, eventually facilitating the translation of bench discoveries to patient-centered therapy.

## 1 Introduction

CRS extends far beyond a chronic sinus infection. It affects up to 15% of the global population and exerts a significant burden on physical health and mental well-being. The impact on quality of life is comparable to major chronic conditions such as congestive heart failure (1, 2). The clinical manifestations mainly include nasal congestion, rhinorrhea, facial fullness or pressure, hyposmia or anosmia, cough, and fatigue, with some patients also experiencing nasal polyps or concurrent lower airway inflammatory diseases such as asthma (3). Phenotypically, CRS is traditionally dichotomized into CRS with (CRSwNP) and without nasal polyps (CRSsNP). Notably, in patients with CRSwNP, non-steroidal anti-inflammatory drug-exacerbated respiratory diseases (AERD) are often associated with more severe nasal polyps and asthma (4). Epidemiological surveys show that the global prevalence of CRS is approximately 5%–15%, with about 30% of CRSwNP patients requiring multiple surgeries due to re-current nasal polyps (5). This relentless chronicity and high recurrence rate, though not directly life threatening, lead to persistent physical and psychological morbidity, encourage antibiotic misuse and increase overall healthcare costs, making CRS a formidable global public health challenge.

**Figure 1:**
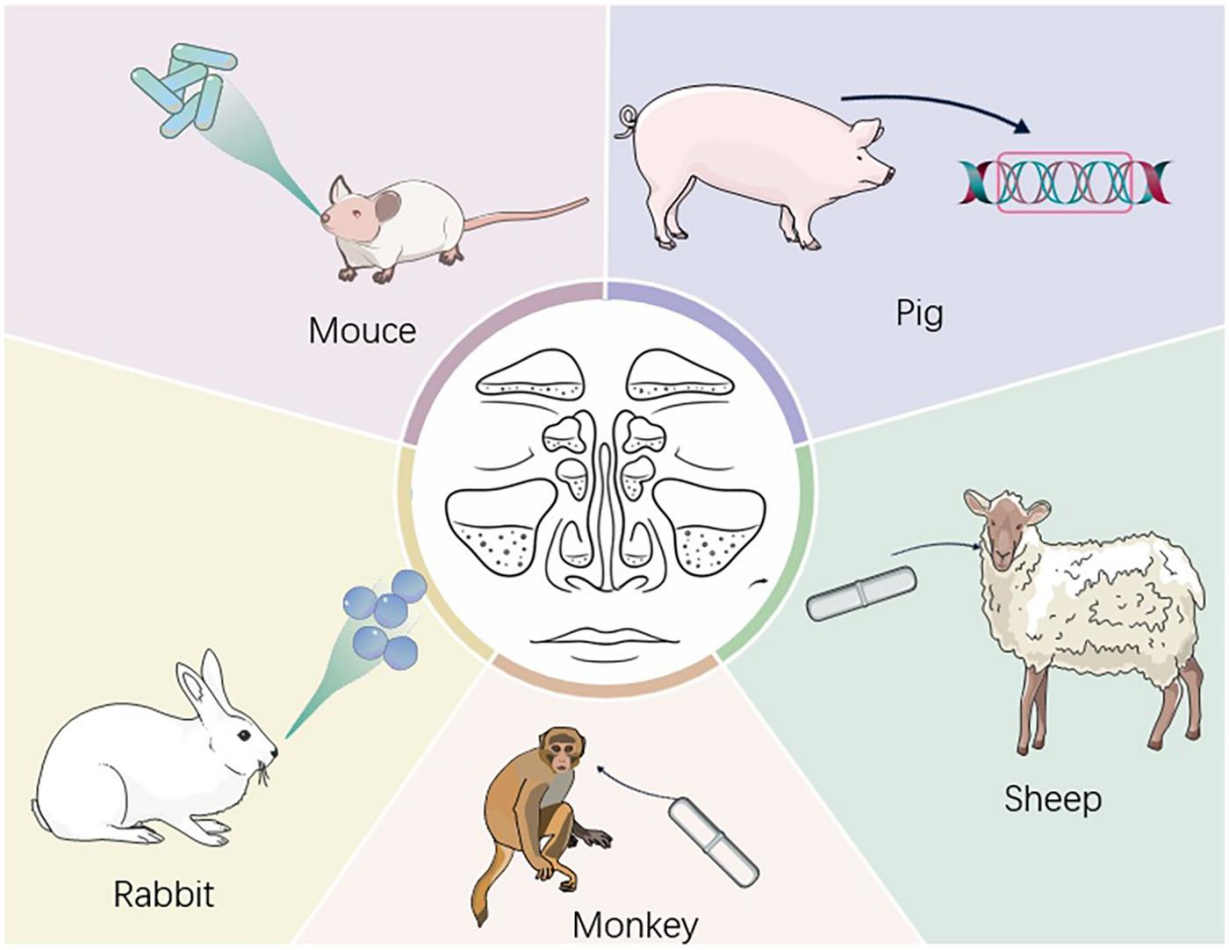
Common animal model types. This figure illustrates the various species utilized to simulate the human sinus environment, highlighting the specific role each species occupies within the field of research. Murine models are employed for investigating underlying mechanisms; rabbits and sheep serve to bridge anatomical disparities; while pigs and non-human primates provide more advanced experimental platforms.

In terms of immune-pathological features, CRS shows significant racial and regional differences. In Western populations, CRSsNP often features neutrophilic infiltration and Th1 cytokine predominance (e.g., IFN-γ), whereas CRSwNP is more often associated with eosinophilic inflammation and Th2-type immune responses (e.g., IL-4, IL-5, IL-13) (1). This Th2-dominant eosinophilic CRSwNP, characterized by severe clinical manifestations and high recurrence after surgery, represents a major challenge in clinical treatment. In contrast to the Western population, neutrophilic inflammation is more common among CRS patients in Asian regions, with a relatively lower proportion of eosinophilic inflammation. While the incidence of eosinophilic CRSwNP in Asia has risen over the past two decades (6), non-type 2 CRS continues to demonstrate a high prevalence and severe disease burden, highlighting the critical limitations of relying exclusively on Th2-focused research models. The etiology of CRS is complex and multifactorial, involving bacterial or fungal infections, allergic reactions, immune deficiencies, and environmental exposures, and is often comorbid with dis-eases such as asthma, aspirin intolerance, bronchiectasis, and cystic fibrosis (2). Despite this, the deep molecular mechanisms underlying CRS development are yet to be fully elucidated, highlighting the importance of establishing reliable animal models. Animal models, with their controllable conditions and high reproducibility, have become indispensable research tools for exploring the pathogenesis of CRS and evaluating new therapeutic strategies. This paper aims to systematically review the pathophysiological features of CRS and the currently used animal models, focusing on the construction methods, applicable scenarios, and limitations of these models, in order to provide theoretical guidance for subsequent mechanism research and therapy development (common animal models of chronic sinusitis are shown in Figure 1).

## 2 Pathophysiology of CRS

### 2.1 Inflammatory subtypes of chronic rhinosinusitis and the evolution of pathological mechanisms

CRS is a highly heterogeneous, multifactorial inflammatory continuum affecting the sinonasal mucosa (1, 7, 8), driven by complex pathogenic mechanisms (8–10). While CRS has traditionally been defined by clinical phenotypes (i.e., the presence or absence of nasal polyps), its characterization has transitioned toward an endotype-driven paradigm, facilitating precision medicine. CRSwNP is classically associated with type 2 (Th2) inflammation characterized by eosinophilia, elevated IL-4, IL-5, and IL-13 levels, asthma comorbidity, and a high risk of recurrence (1, 7, 8). However, accumulating evidence reveals broader pathophysiological complexity. Some patients exhibit mixed Th1/Th17 activity, neutrophilic infiltration, and extensive tissue remodeling (e.g., subepithelial fibrosis and basement membrane thickening) (8). High-resolution technologies, particularly single-cell transcriptomics, have further delineated these inflammatory landscapes into distinct molecular endotypes (e.g., pure type 2 vs. mixed type 1/3) that dictate clinical trajectories and therapeutic responses (10–12). For instance, mixed type 1/3 CRSwNP is generally associated with earlier relapse and a poorer response to conventional type 2-targeted therapies (8, 11). Ultimately, the application of these molecular and immunological endotypes constitutes a critical and logical framework for accurate prognostication and individualized therapeutic decision-making for the future management of CRS (1, 8, 9).

### 2.2 The pathological core: barrier failure, immune stalemate, and tissue remodeling

The clinical persistence of CRS is driven by a self-perpetuating triad consisting of epithelial barrier failure, immune hyper-reactivity and irreversible tissue remodeling (1, 7). This phenomenon can be explained by the “two-hit” hypothesis. In this hypothesis, a first genetic or environmental insult to the mucosal barrier predisposes the epithelium to a maladaptive response to subsequent microbial triggers. This barrier damage facilitates pathogen colonization (8, 13), especially bacterial biofilms which are resistant to clearance and create a persistent reservoir of chronic inflammation (14, 15).

Barrier disruption, followed by aberrant immune activation, sustains the disease. Type 2 CRS is characterized by recruitment of Th2 cells, eosinophils and ILC2s, which is amplified by epithelium-derived alarmins (TSLP, IL-25, IL-33) (7, 8). In contrast, non-type 2 CRS is characterized by neutrophils, Th1/17 cells, and increased IFN-gamma and IL-17 (11). Constitutive activation of intracellular signaling pathways (e.g., NF-κB, JAK-STAT, PI3K/AKT) by this chronic inflammation suppresses anti-inflammatory mechanisms and blocks spontaneous resolution (8, 16).

Chronic inflammatory stimuli therefore slowly lead to structural changes such as basement membrane thickening, stromal edema and polyp formation (7). TGF-β is a key regulator of eosinophilic infiltration and epithelial-mesenchymal transition (EMT) in type 2 inflammation (7, 16). Indeed, this tissue remodeling is strongly linked to disease severity and the risk of post-operative recurrence (17).

Current biologics are effective in attenuating inflammation but are unable to reverse fibrosis or EMT. Therefore, future CRS studies and disease models need to focus on both the immune signature and structural repair, e.g., by targeting the TGF-β signaling, to achieve true disease modification (1, 18–20).

## 3 History and development of animal models

### 3.1 Origins of sinusitis animal models

The formalization of sinonasal modeling originated in the 1960s, catalyzed by Messerklinger's seminal observations that linked mechanical obstruction to ensuing mucosal inflammation-a paradigm that has dictated the CRS research trajectory for over half a century (21). Since then animal models have been considered as indispensable research tools towards defining etiology and pathophysiology of CRS. With increased appreciation of CRS as a heterogeneous and multi-factorial illness, there has been increased utility of these models and they have become critical platforms on which the mechanistic inquiry and preclinical testing of new therapeutic measures may be conducted.

### 3.2 Evolution of modern animal models

Existing preclinical modelling of CRS is becoming strategically characterized by a trade-off between experimental tractability and translational fidelity.

Although murine models (in particular excellently suited Th2-prone BALB/c and Th1-dominant C57BL/6) are still the essential “mechanistic engines” in mucosal immunology, they are limited in use by a deeper evolutionary split (22). The experimental convenience of genetic manipulation is counterbalanced by inherent anatomical constraints: the absence of true maxillary sinuses and their primitive mucociliary mechanism are a serious anatomical constriction. Accordingly, a drug candidate, which is able to suppress eosinophilia in mice, can be inert with a human endotype of heterogenous Th17/fibrotic remodeling.

In contrast, bigger mammals like rabbits and sheep are used to fill the anatomical gap, providing sinuously shaped architecture and drainage dynamics with which human physiology is closely recapitulated (23).

Emerging frontiers involving porcine and non-human primate (NHP) models offer the most sophisticated immunological repertoires and tissue remodeling capacity to date. However, their integration into mainstream research is constrained by ethical concerns, prohibitive overhead, and a relative scarcity of species-specific molecular probes. Ultimately, the selection of a CRS model must transcend convenience; it requires a rigorous alignment between the model's biological ceiling and the specific clinical hypothesis-be it cytokine-driven inflammation, post-surgical fibrosis, or barrier dysfunction.

### 3.3 Mouse models

#### 3.3.1 Advantages of mouse models

The most common experimental models used in basic research in CRS are mice because of their well-defined genetic background, short reproductive cycles, standardized protocols and relatively low maintenance costs. Notably, this is because the nature of the latest genetic engineering technologies, such as gene knockout, knock-in, and transgenic methods make murine models indispensable in mechanistic studies. These have allowed precise dissection of the functions of individual inflammatory mediators, intracellular signaling pathways, and immune cell subsets, thereby providing significant contributions to current knowledge of the molecular and immunological pathways that mediate CRS pathogenesis.

#### 3.3.2 Murine induction strategies

Because murine models lack spontaneous sinonasal chronicity, diverse induction strategies have been developed to accurately reproduce different CRS endotypes.

- Allergen-Induced Models: Allergen-induced murine models are designed to mimic type 2 immune responses through repeated intranasal administration of allergens, most commonly ovalbumin (OVA) or house dust mite (HDM). These models usually cause eosinophil-driven inflammation with hyperplasia and excessive secretion of mucus in the glands; hence, they are especially appropriate in the study of eosinophilic CRS (ECRS). To further augment the severity of inflammation and nasal polyp-like formation, other agonists are often added including staphylococcal enterotoxin B (SEB) or fungal protease (24, 25). Efforts to induce chronic phenotypes from these transient states have recapitulated “polyp-like” mucosal thickening, most notably through intensive protocols combining HDM, Aspergillus, and SEB over an extended 16-week period (26, 27). Therefore, it is most suitable for investigating the pathogenesis of Type 2 CRS (ECRS) and for screening biologics targeting Th2 cytokines.
- Bacterial and fungal infection models Infection-based disease models in mice, such as those using Pseudomonas aeruginosa, Alternaria, or Staphylococcus aureus, are designed to define the microbial triggers of sinonasal inflammation. These strategies have evolved from simple intranasal instillation to more complex persistence strategies, the most notable being the use of alginate-encapsulated P. aeruginosa to escape rapid murine clearance and maintain neutrophilic infiltration (28, 29). Repeated (14 days) exposure to S. aureus exotoxins has also been effective in inducing lymphoplasmacytic signatures in rat models (30). Interestingly, fungal challenges with Alternaria have been shown to induce a Th2-biased endotype, breaking the classic dichotomy between strictly infectious and strictly allergic paradigms (22). However, these infection models have a translational dilemma: they model largely acute pathogenic invasion, rather than the low-grade, maladaptive microbial dysbiosis seen in human chronic disease. Therefore, these platforms are better suited to test novel antimicrobial therapies, study mechanisms of biofilm formation, and probe antimicrobial resistance in non-type 2 inflammation, rather than modelling persistent chronic dysbiosis.
- Chemical Stimulation Models: Models based on chemicals (mainly using lipopolysaccharide (LPS) or phorbol myristate acetate (PMA) offer an efficient, simple platform by which sinonasal inflammation can be elicited by direct activation of either the TLR4/NF-κB or protein kinase C axes (31). The logistic ease of these systems makes them viable for a volume of screening of new anti-inflammatory moieties; instances are the IKKε/TBK1 inhibitor Amlexanox which exhibited considerable suppression of pro-inflammatory cytokines in the LPS-challenged murine mucosa (32). Precisely for this reason, such models are recommended solely for reductionist pharmacological studies (e.g., verifying whether a specific drug can block a particular molecular signaling node).
- Genetically Modified Models: Genetically modified murine strains-leveraging constitutive knockout, knock-in, or cell-specific conditional systems-represent the vanguard of mechanistic dissection in CRS. These models have revealed the key pathways of mucosal breakdown through the isolation of discrete molecular variables. One of them is the epithelial-centric Sprr2a−/− model that has shown that the absence of this stress-response protein increases inflammation at OVA/SEB induced, and catalyzes epithelial-mesenchymal transition (EMT) via the SAA2-signaling pathway (33). In the same line, studies of Nrf2 deficient mice have highlighted the importance of redox homeostasis in that the impaired antioxidant defenses of the sinonasal mucosa lead to disposition to environmental stressors (34). They should be viewed as high-resolution lenses for specific pathways, applicable to the functional validation of key pathogenic genes, provided they are combined with environmental triggers.
- Surgical Models: CRS surgical paradigms which require physical obstruction of sinonasal outflow tracts through the aid of absorbable packing aim to reiterate the so-called ventilation-drainage failure considered historically as the sine qua non of the sinonasal disease. In combination with immunogenic stimuli (e.g., OVA or SEB), such models are capable of producing heterogenous granulocytic infiltrates, hypertrophy of the mucosa, and aberrant osteogenesis (35). Initial studies that apply post-obstruction inoculation of Bacteroides fragilis have emphasized the role of stasis in supporting bacterial persistence (36), and BALB/c strains have also shown to have an enhanced eosinophilic remodeling in response to similar hypoxic stress (37). A functional osteomeatal complex (OMC) is found in the murine sinonasal architecture, which involves shallow recesses in place of discrete maxillary sinuses (38). Because of these distinct architectural features, the usefulness of surgical models is most limited to the interrogation of local pharmacokinetics or hypoxia-induced cytokines in a microenvironment.

#### 3.3.3 Limitations of murine models

Although murine models of CRS are logistically tractable, there is a longstanding translational paradox in their utilization: murine models are effective for elucidating the discrete mechanistic components of the disease in a reductionist manner but are limited in recapitulating the integrated pathophysiology of the human disease. Such disconnection is attributed to four underlying constraints:

Anatomical Incommensurability: The murine lack of real paranasal sinuses, replaced by shallow and non-pneumatized recesses that have no OMC confines experimental obstruction to an effect of the nasal cavity and not the reenactment of mural outflow failure.

Immunological Oversimplification: The human CRS landscape is a complex mosaic of Th2, Th17, and ILC2-driven endotypes, localized IgE production within a single polypoid landscape is common. By comparison, murine models mostly impose monotypic polarization, not reflecting the low-grade milieu of refractory CRSwNP.

Technical Artifice: The natural initiation of the disease is circumvented by depending on supraphysiological inductions, either exogenous superantigens or induced deletions of endogenous genes. Such generated phenotypes are often acute and reversible, which stands in contrast to the chronic epithelial barrier breakdown that has been accepted as the instigating factor of clinical chronicity.

Interspecies Divergence: Divergence in immune responses, drug metabolism, and tissue remodeling can cause translational limitations as a result of species differences.

Finally, the search of a flawless model of the mouse should be substituted with a system of intentional choice. Such translation plasticity is only possible by aligning model architecture with targeted pathobiological investigation: using allergen models to target Th2 biologics, barrier-disruption models to repair epithelial, and integrated two-hit model platforms to polygenically validate. The future advancements depend on the focus on biological relevance rather than convenience of the experiments.

### 3.4 Rabbit models

#### 3.4.1 Advantages of rabbit models

The use of rabbits in CRS studies is largely based on their anatomical analogy to humans, particularly regarding their nasal cavity structure, maxillary sinus dimensions, and mucociliary activity. These features make rabbit models particularly useful in investigations where surgical manipulation, sinuosity of obstruction, radiology and local drug delivery are necessary. Consequently, the rabbit has emerged as the gold standard for interventional and pharmacokinetic evaluations. Their physical dimensions facilitate repeated endoscopic access, serial sinus lavage for biomarker longitudinal profiling, and the deployment of drug-eluting stents or biodegradable implants under high-resolution CT or MRI guidance.

#### 3.4.2 Modeling methods and applicability

The developmental trajectory of rabbit CRS models mirrors a sophisticated transition from capturing acute infection to simulating the self-sustaining mucosal failure of chronic disease. Early paradigms such as that by Marks (1997) with S. pneumoniae impregnated sponges, had been able to induce acute purulent exudation but not to transition to chronicity. This pilot failure highlighted a basic physiological fact, namely exogenous bacterial challenge in isolation cannot overcome the strong mucociliary clearance (MCC) of the rabbit in the absence of the initial stimulus (39). To overcome this mucociliary clearance threshold, later procedures substituted it with mechanical obstruction of the ostia, which used the high anatomical similarity of rabbit maxillary sinus to human maxillary sinus, to impair physiological mucociliary drainage, and persistent microbial colonization (40). However, the definitive breakthrough in modeling true CRS chronicity was achieved by Liang et al., who successfully decoupled the initiation of inflammation from long-term maintenance (41). They were able to create a two-hit pathophysiological state (submucosal PMA injections, as a surrogate of epithelial alarm signaling, followed by a transient unilateral Merocel occlusion) to accomplish a two-hit pathophysiological state. Notably, the clinical temporal requirements of CRS are met by this model, and it sustains inflammation more than 12 weeks after obstruction removal. This indicates that a brief mechanical trauma, when overlaid over an already activated immune background, has the potential to reprogram the mucosal niche on a permanent basis.

However, the use of PMA, which is a strong, non-specific agonist of PKC, creates the dissonance of immunological response to some extent. The resultant cellular inflammation is largely neutrophilic and macrophage-mediated with an inability to replicate the lymphoplasmacytic structure or B-cell follicular pattern which is core to most human CRSwNP endotypes. Consequently, while the Liang model is a powerful tool for investigating innate-mediated remodeling, it falls short in its clinical relevance to the adaptive immune pathways that characterize chronic, recalcitrant disease in humans.

#### 3.4.3 Suitability and limitations

Utilization of rabbit CRS models is not universal but is occupied by a discrete set of functional niches determined by its adherence to particular dimensions of pathology. To choose a rabbit paradigm, a sub-profile needs a delicate conformity of the biological basis of the model with the hypothesis of the clinical application:

- Sponge-induced Obstruction: Offers an aerodynamic platform of investigating mucosal responses to acute bacterial insult. But its non-persistent form cannot reflect the immune dysfunction and tissue remodeling that drive clinical chronicity.
- Surgical Ostial Closure via Bacterial Inoculation: Best longitudinal imaging and pharmacokinetic (PK) assessments. However, the surgery trauma required creates confounding cascades of wound-healing that can be enough to obscure endogenous inflammatory pathways of CRS.
- PMA-driven Chronic Models: A high step forward in the field of temporal persistence (>12 weeks). However, they are innate-centric, neutrophilic dominated, macrophagic dominated with a consistent failure to produce the edematous eosinophilladen polypoid features of human CRSwNP. This difference is probably due to inherent interspecies differences in epithelial-immune crosstalk.

Overall, rabbit models are important in CRS studies, especially on the anatomical, im-aging and interventional studies. Nevertheless, their shortcomings in complete rein-statement of immunological heterogeneity and chronic progression of human CRS makes them require sound use of models in accordance with desired research objectives.

### 3.5 Sheep models: surgical and biofilm standards

Sheep have sinonasal anatomy found in humans which are similar in sizes, space orientation, and draining outlets to allow them to be used in translational studies on surgery (42). In addition, sheep naturally develop conditions such as parasitic infestation (e.g., by the sheep nasal botfly), which causes eosinophilic sinusitis and clinical manifestations which include nasal discharge (43, 44). The generation of these spontaneous pathologies offers a special benefit to the modeling of particular CRS endotypes, specifically eosinophilic inflammation. Ovine CRS models utilizing Staphylococcus aureus effectively recapitulate the biofilm-associated infectious states seen in humans. Notably, the use of nanoparticles to target alpha-toxin in these models has shown efficacy in inhibiting biofilms, providing a robust platform for testing next-generation antimicrobial therapies (45). Sinus ostia are also blocked by mechanical obstruction and foreign-body implantation practices leading to lasting local inflammation, thickening of the mucosa and so forth. Such models have come in especially handy when studying the sinus physiology, biofilms persistence, and long-term inflammatory remodeling (46).

Sheep models are limited to practicability, in spite of their advantages as a translation tool. They are large, which raises housing and procedural expenses and again limits the sample sizes and the throughput of the experiments. Also, there are more stringent ethical and regulatory requirements in research involving large-animals, which require special facilities and expertise. As a result, although sheep models can be of high importance in surgical simulation, biofilm research, and testing preclinical therapeutic, their deployment must be under close consideration on the basis of the re-search aims, cost-effectiveness, and the possibility of ethics.

### 3.6 Non-human primates: immunological proxies

Emerging animal models are now being guided toward realistic recapitulation of the complicated pathological processes of CRS in humans, as well as improving their merit as translational tools. These include, among others, non-human primate models which offer the most promising avenue in terms of evaluating the safety, efficacy, and pharmacodynamics of biologic agents and a surgical intervention due to their close resemblance to humans in terms of immune system structure, sinonasal anatomy and disease progression (47).

The latest developments are a patented mechanical obstruction method (WO/2025/118983) whereby expandable sponges are inserted into the nasal cavity to create a lasting obstruction. This strategy has been reported to induce an inflammatory response on the mucosa, polypoid changes, and remodeling of tissues in the long term, which are strong resemblances to the main pathological features of human CRS. These types of models provide a previously unparalleled chance to examine disease etiology and therapeutic responses within an environment that closely approximates clinical reality (48). However, constrained by extremely stringent ethical regulations, exceedingly cumbersome logistical management procedures, and prohibitively high costs, such models remain difficult to apply in the majority of routine mechanistic exploratory studies.

### 3.7 Porcine models: the CFTR paradigm shift

CFTR-knockout pigs represent a paradigm shift, manifesting spontaneous, lesion-specific CRS. They faithfully recapitulate the “vicious cycle” of viscous mucus stasis, ion transport abnormalities, and ciliary arrest-making them peerless for testing CFTR modulators. The models are the only ones that closely mimics the natural disease progression of CRS and gives a robust platform to study potential CFTR-targeted therapies and pathogenesis underlying it (49).

However, large animal models are still lacking in extensive application due to strict ethical requirements, expensive maintenance and complex operation. In the case of porcine models, the development of spontaneous chronic lesions requires a long latency period, which substantially increases the time and financial investment required for the experiment (50, 51).

### 3.8 Comparative analysis and model selection framework

#### 3.8.1 The universal problem of interspecific divergence

No non-human system can perfectly mimic the complex “mosaic” of human CRS endotypes. Interspecies divergence is a universal constraint, whether it is the absence of a true osteomeatal complex (OMC) in mice, the predominantly innate-driven neutrophilic response in rabbits, or the highly specialized mucosal immunity in sheep. Additionally, an important factor in clinical criticism is the temporal mismatch between the rapid induction of disease possible in animal models (weeks to months) and the longstanding chronicity of human CRS, which can last for decades. Thus, these experimental platforms should be seen as pathobiological proxies and not as perfect human surrogates.

#### 3.8.2 Model selection strategy guide

In order to bridge the remaining translational gap, researchers need to move away from convenience-based animal selection to an endotype-driven approach. The choice should be rational and motivated by the specific pathobiological hypothesis under test:

Mechanistic Discovery and Genetic Pathway Analysis Murine models are the undisputed gold standard for the identification of discrete molecular variables or the interrogation of specific inflammatory circuits (e.g., Type 2 inflammation). Their advanced gene editing tools enable high-throughput high-resolution dissection of immune-epithelial crosstalk.

Surgical Simulation and Targeted Drug Delivery: Rabbit and sheep models are optimal where topographical realism and sinonasal pharmacokinetics are important. Due to their human-like paranasal dimensions and mucociliary clearance, they are indispensable for assessing endoscopic interventions and the mucosal healing kinetics of drug-eluting scaffolds.

Biofilm Dynamics and Chronic Dysbiosis: Ovine (sheep) models following Staphylococcus aureus are effective in recapitulating biofilm-associated infectious states for the study of persistent microbial dysbiosis. CFTR-knockout pig models are also uniquely suited to study spontaneous, viscous mucus stasis.

Preclinical Biologic Vetting: The model with highest translational fidelity for evaluating the safety, pharmacodynamics, and efficacy of new biologics targeting refractory or mixed endotypes is the non-human primate (NHP) due to the unique similarity of NHP and human immune architecture. Common methods for creating animal models are shown in Table 1.

**Table 1:** Summary of validated modeling protocols and experimental outcomes across animal species.

Author	Animal	Material	Surgery	Time	Model
Lindsay et al. (52)	BALB/c mice	A. fumigatus	No	12 weeks	ECRS
Kim et al. (24)	BALB/c mice	OVA + SEB	No	8 weeks	ECRSwNP
Kim et al. (53)	BALB/c mice	OVA + Aspergillus protease	No	8 weeks	CRSwNP/ECRS
Khalmuratova et al. (27)	C57BL/6 mice	HDM + SEB	No	8 weeks	ECRSwNP
Kim et al. (25)	C57BL/6 mice	AP + OVA	No	12 weeks	ECRS
Bae et al. (54)	ApcMin/+ mice	OVA + SEB	No	12 weeks	CRSwNP
Ramanathan et al. (34)	Krt5-cre mice	HP	No	10 days	ECRS
Cha et al. (55)	Sirt5−/− homozygous mice	OVA + SEB	No	12 weeks	ECRS
Sun et al. (56)	uPA−/− mice	Merocel nasal packing + S. aureus	No	12 weeks	CRSsNP
Migliavacca Rde et al. (57)	New Zealand white rabbit	Merocel nasal packing	Yes	12 weeks	CRSsNP
Jia et al. (58)	New Zealand white rabbit	SEB	Yes	4 weeks	CRSsNP
Liang et al. (41)	New Zealand white rabbit	PMA + Merocel nasal packing	Yes	12 weeks	CRSsNP
Boase et al. (59)	Sheep	A. fumigatus; A. alternata; S. aureus	Yes	8–12 weeks	CRSsNP
Ha et al. (44)	Sheep	S. aureus	Yes	8 weeks	CRSsNP
Zhang and Zhang (48)	NHP	Merocel nasal packing	No	12 weeks	CRS
Rogers et al. (49)	Pig	CFTR knockout	No	3 weeks	CRS

This table summarizes representative CRS modeling protocols, including species, induction materials, surgical interventions, observation periods, and resulting pathological phenotypes. The table highlights murine ECRS and CRSwNP-like models, obstruction-based rabbit and sheep models, and emerging NHP and porcine platforms.

#### 3.8.3 Toward an intelligent modeling framework

To realize the full potential of these platforms, future investigations need to progress towards an Intelligent Modelling framework that pairs the architectural advantages of specific models with specific clinical questions:

Refining Induction: Developing “two-hit” systems (e.g., combining genetic susceptibility with mechanical stasis) to shorten modelling time without sacrificing pathological accuracy.

Multi-omics Integration: We integrate single-cell RNA sequencing (scRNA-seq) data from multiple species to map the pathogenetic trajectory of CRS, linking descriptive histology to molecular immunology.

Combination Validation: Moving beyond testing monotherapy to multi-modal approaches such as combinations of novel biologics with localised drug-eluting scaffolds.

Final Consistent Statement: Better than any other model there is no model. The validity of an animal study is contingent upon the strategic fit between the biological architecture of the model and the specific pathobiological hypothesis being tested. Rodent models will remain indispensable for reductionist pharmacology and genetic pathway analysis, but large animal and non-human primate platforms are needed to validate translational interventions for which rodent evidence is insufficient. Ultimately, progress will come from focusing on clinical relevance rather than experimental convenience. The characteristics of various animal models of CRS are shown in Table 2.

**Table 2:** Comparative synthesis of animal model utility: strengths, translational constraints, and strategic applications.

Animal model	Key strengths	Target subtypes	Limitations	Research focus
Mouse models	Low cost; rapid breeding; defined genetic background; mature gene-editing tools; suitable for high-throughput mechanistic studies	ECRS; neutrophilic inflammation; CRSwNP-like and CRSsNP-like models	Major anatomical differences from humans; no single model captures full CRS complexity; surgical models are demanding and variable; species-related translational gap	Molecular pathway mapping; immune-cell profiling; drug-target screening and validation
Rabbit models	Human-like maxillary sinus anatomy; enables surgical manipulation, imaging, and longitudinal assessment	CRS models; acute/subacute inflammation; obstruction-based models	Classical surgical models involve trauma and may not sustain chronicity; modified models are non-spontaneous and usually lack typical nasal-polyp formation	Surgical technique evaluation; local drug delivery; imaging-based outcome metrics
Sheep models	High anatomical similarity to humans; large sinus volume supports refined surgical manipulation and long-term implant studies	Eosinophilic CRS; biofilm-associated CRS; chronic infection models	High cost; strict husbandry; complex ethical review	Biofilm formation and therapy testing; surgical training; implant materials and local sustained-release drug evaluation
Novel animal models	NHP: closest immune and anatomical approximation to humans; useful for biologic safety and efficacy evaluation. Pig: gene-edited CFTR models support spontaneous cystic-fibrosis-related CRS modeling	NHP: complex immune-mediated CRS. Pig: etiology-specific CRS, including cystic-fibrosis-related disease	NHP: strict ethics, very high cost, and complex handling. Pig: long study duration and high housing cost	Translational studies; complex pathophysiology modeling; preclinical evaluation of advanced biologics and gene therapies

This table compares predictive validity and experimental tractability across animal platforms. Mice provide mature gene-editing tools and low cost but have major anatomical limitations. Rabbits and sheep are useful for surgical simulation and local drug-delivery studies because of their larger sinonasal anatomy. NHP and porcine models offer higher translational fidelity for selected immunological or etiology-specific questions, but cost, ethics, and logistics restrict their use.

## 4 Core value of animal models in CRS mechanistic research and clinical translation

Animal models play a critical role in CRS studies with a role in providing essential insights into both the molecular pathogenesis, identification of treatment targets and preclinical testing of new interventions. These models provide an experimental platform through recapitulation of the major pathophysiological events, such as inflammatory cascade hallmarks, dysfunction of inflammatory epithelial structures, tissue remodeling, and nasal polyp development. Current studies are now moving beyond the descriptive anatomy stage of research mechanistic platforms that combine CRISPR-based editing with single-cell transcriptomics to map endotype-specific pathways. Ultimately, the metric for model success must shift from mere anatomical mimicry to predictive validity, positioning these preclinical models become a strict filter of the therapies that are to undergo human testing.

### 4.1 Target identification: beyond correlation to causal discovery

Animal models have evolved beyond phenotypic observation, becoming critical tools for uncovering therapeutic nodes that were previously obscured by clinical heterogeneity. To illustrate, epithelial fragility—rather than sheer inflammatory burden—is a primary driver of EMT; this non-inflammatory process was found through CRISPR/Cas9-mediated depletion of Sprr2a in an OVA/SEB-sensitized mouse (33). Similarly, utilizing a murine model of cigarette smoke-induced oxidative stress, researchers identified the PI3K/AKT/NF-κB cascade as a master regulatory hub for tissue remodeling, which is also subject to natural modulation by osthole (60). Altogether, these studies demonstrate that highly tailored animal models do not only re-capitulate discrete CRS endotypes, but also provide the ability to screen and confirm therapeutic candidates systematically, including both inflammatory effectors and important signaling centers.

### 4.2 Decoding the pathogenic niche: epithelial-immune-microbial triads

Multimodal animal models provide alternative ways of interrogate the multifaceted pathogenic mechanisms of CRS. The BALB/c mice that were allergic to Alternaria showed that IL-4/IL-13 coordinated mucosal recruitment by up-regulating epithelial CCL11, and not by enhancing the strength of primed signals (22). In contrast, models that are genetically modified allow the dissection of single molecular functions comprehensively and in a granular fashion. Parallel investigations into mucosal instigations in uPA-knockout mice have implicated the Wnt β-catenin axis as a stabilizer of the mucosal barrier in acute inflammatory initiation (56). Canonical inflammatory conduits, including NF-κB catenin, MAPK and JAK/STAT, can be prompted by chemical stimulations, using LPS or PMA, which provides a special insight into the beginning of CRS (61). These complementary strategies come together to build a combined structure in the characterization of cytokine networks, epithelial immune crosstalk and signal transduction cascades in CRS pathogenesis. However, no model of low-grade, polymicrobial dysbiosis of human disease has been developed in the field.

### 4.3 Translational screening and tiered validation

Addressing the translational gap necessitates a tiered validation strategy, where murine models act as initial filters to evaluate small-molecule candidates (e.g., quercetin, amlexanox) targeting the STAT3/6-driven EMT pathway (32, 62). Nevertheless, anatomical scale is the dominant factor of success in case of localized delivery modalities such as stents, hydrogel or nanoparticle carriers. Mucosal retention kinetics and barrier restoration [e.g., thymoquinone-mediated p63/claudin upregulation (63)] can only be easily studied in rabbit and rat surgical models, despite being imperfect sinonasal homologues, these models are essential for evaluating it. These models highlight the broad applicability of ani-mal models in the therapeutic vetting model, whether natural extracts or direct bio-logics, which in turn provides a solid basis of empirical evidence on clinical translation.

### 4.4 Future directions in CRS animal model development

Bridging the bench to bedside gap requires strengthening the links between animal models and human data. Success of models should no longer be measured by mimicking anatomy alone, but by predictive validity, which is a stringent test for therapies in human trials.

Future CRS animal models should be more precise, heterogeneous and clinically translatable to faithfully recapitulate disease complexity and support personalised treatment paradigms. A very promising approach is the use of gene-editing technologies such as CRISPR/Cas9 to activate or suppress specific inflammatory cascades or molecular effectors, recapitulating faithfully a variety of CRS endotypes to allow mechanistic understanding and target validation (34).

At the same time, we should promote cross-species and multimodal integrative approaches. By combining the genetic tractability of mouse models with the anatomical and surgical relevance of larger species (e.g., rabbits, sheep) and the multidimensional data from histopathology, immunology, imaging and omics, composite model systems can be generated that are more clinically relevant for comprehensive evaluation of interventions (38).

Clinical calibration can also be achieved by the application of scRNA-seq and multi-omics, and by direct comparison of models with patient samples. The link between descriptive histology and molecular immunology, as well as clinical phenotypes, imaging aspects and dynamic biomarkers in evaluation models, will tremendously improve their predictive power for treatment response and counselling individualised therapeutic approaches (64).

## 5 Conclusions

The usefulness of animal models in the study of CRS has evolved beyond information about the anatomy to the mechanism-explainable emulation. As this review highlights the choice of the model needs to be based on evidence of endotypic halting other than chance of experimentation convenience. Although murine systems are the gold standard of Type 2/ILC2 circuitry dissection, their hegemony has unintentionally limited the therapeutic focus to eosinophilic patterns, e.g., at the expense of the nearly 50% of CRSsNP cases of non-type 2 inflammation, a critical translational blind spot given the rising prevalence and severe disease burden of neutrophilic, non-type 2 CRS, particularly within Asian populations.

Higher mammals (rabbits, sheep, pigs) have a critical niche in which topographical realism and sinonasal pharmacokinetics are the key success factors. However, their immunological tractability is limited, and neither the existing species spontaneously or recapitulates the architectural signature of human polyposis, e.g., basement membrane thickening or massive stromal edema, which are often hallmarks of mixed or non-type 2 refractory disease.

The shift of the field in the endotype-oriented modeling is long overdue. Nevertheless, we can no longer live by the eosinophil-neutrophil dichotomy. New single cell atlases now describe at least four reproducible CRS endotypes with each characterized by a unique epithelial-immune crosstalk signature. Incorporating such particular molecular states other than just cellular infiltrates should be the goal of future models.

The price of translational disengagement is great: the collapse of early IL-5 trials in unselected patients-even with strong activity in Th2-polarized murine models-offers a costly lesson to the danger of disregarding endotypic limits. Predictive accountability is the future of CRS modelling. The key to success is to make sure that the biological ceiling of a model reflects the group of patients that needs a particular therapy. It is only in this way that preclinical breakthroughs can result in stratified as opposed to serendipitous clinical success.

As genetic engineering advances further, multi-omics approaches, and strategies of translational models, animal models are becoming more competent to recapitulate the complex and dynamic pathophysiological attributes of CRS. The developments will further consolidate the distance between clinical application and basic research. Finally, rational selection and strategic integration of suitable animal models will be-come a decisive factor in enhancing CRS mechanistic knowledge and hastening the process of translating findings of experimental studies into viable, individualized treatments.
